# Temporal change of photophobic step-up responses of *Euglena gracilis* investigated through motion analysis

**DOI:** 10.1371/journal.pone.0172813

**Published:** 2017-02-24

**Authors:** Kazunari Ozasa, June Won, Simon Song, Shun Tamaki, Takahiro Ishikawa, Mizuo Maeda

**Affiliations:** 1 Bioengineering Lab, RIKEN, Wako, Saitama, Japan; 2 Department of Mechanical Convergence Engineering, Hanyang University, Seongdong-gu, Seoul, Korea; 3 Institute of Nano Science and Technology, Hanyang University, Seongdong-gu, Seoul, Korea; 4 Department of Applied Bioscience and Biotechnology, Faculty of Life and Environmental Science, Shimane University, Matsue, Shimane, Japan; 5 Core Research for Evolutional Science and Technology (CREST), Japan Science and Technology Agency (JST), Chiyoda-ku, Tokyo, Japan; US Naval Research Laboratory, UNITED STATES

## Abstract

The adaptation to a strong light is one of the essential characteristics of green algae, yet lacking relatively the information about the photophobic responses of Eukaryotic microalgae. We investigated the photophobic step-up responses of *Euglena gracilis* over a time course of several hours with alternated repetition of blue-light pulse illumination and spatially patterned blue-light illumination. Four distinctive photophobic motions in response to strong blue light were identified in a trace image analysis, namely on-site rotation, running and tumbling, continuous circular swimming, and unaffected straightforward swimming. The cells cultured in autotrophic conditions under weak light showed mainly the on-site rotation response at the beginning of blue-light illumination, but they acquired more blue-light tolerant responses of running and tumbling, circular swimming, or straightforward swimming. The efficiency of escaping from a blue-light illuminated area improved markedly with the development of these photophobic motions. Time constant of 3.0 h was deduced for the evolution of photophobic responses of *E*. *gracilis*. The nutrient-rich metabolic status of the cells resulting from photosynthesis during the experiments, i.e., the accumulation of photosynthesized nutrient products in balance between formation and consumption, was the main factor responsible for the development of photophobic responses. The reduction-oxidation status in and around *E*. *gracilis* cells did not affect their photophobic responses significantly, unlike the case of photophobic responses and phototaxis of *Chlamydomonas reinhardtii* cells. This study shows that the evolution of photophobic motion type of *E*. *gracilis* is dominated mainly by the nutrient metabolic status of the cells. The fact suggests that the nutrient-rich cells have a higher threshold for switching the flagellar motion from straightforward swimming to rotation under a strong light.

## Introduction

Motile microalgae such as *Euglena gracilis* and *Chlamydomonas reinhardtii* are fascinating organisms, because they have a high photosynthetic capacity and are able to move using their flagella. There is a large body of research on exploiting their photosynthetic capability to produce bio-fuels, pharmaceuticals, or nutritious substances in a labour-, cost-, and facility-effective and environmentally friendly manner [1–5]. For instance, higher oil production has been achieved by modifying genes in *C*. *reinhardtii* [6,7] and *E*. *gracilis* [8,9], and the development of appropriate media has allowed *E*. *gracilis* to be cultured on a pond-scale [10]. Another potential use of motile microalgae is in cell-based biosensors. Such biosensors could be used to monitor and detect environmental toxicity and/or to screen for drug side-effects based on the chemotactic responses of cells to environmental substances [11,12]. Biosensors using microalgae cells confined in a microfluidic chip have several advantages over similar cell viability tests with mammalian cells in a culture dish [13]. That is, it is faster and easier to measure the locomotive responses of motile microalgae cells than to measure changes in the viability of mammalian cells. Another challenging application is to develop natural soft-computing based on the photophobic responses of motile microalgae cells [14–16]. Simple neural computing has been demonstrated with *E*. *gracilis* cells by using a neural network algorithm and subjecting the cells to optical stimuli [17,18]. That study demonstrated that computational performance could be improved by the various photophobic responses of the microalgae cells.

One major issue in the above applications is that the cells’ characteristics change over time. That is, a certain group of cells will respond differently over time because of spontaneous cell growth, and/or changes in the environment or external stimuli. For example, even when cells have shown strong chemotactic sensitivity to a certain substance, their sensitivity will weaken over time, resulting in decreased performance and poor reproducibility of chemical biosensing. Another issue is wide cell-to-cell variations; that is, different responses among cells. Although diversity is an important strategy for cell survival in harsh environments, the differences among cells in their photosynthetic rate or responses to external stimuli can result in performance fluctuations in many applications.

To date, there has been little research on temporal variations in the photophobic responses of microalgae cells. The basic photophobic responses of *E*. *gracilis* have been investigated for many years, and it is well known that strong blue light causes their movement to change from straightforward swimming to random tumbling as the light step-up response [19–22]. Also, the circadian rhythm strongly affects the straightforward swimming speed of *E*. *gracilis* [23,24], i.e., their swimming speed increases during their subjective daylight time. Although many microalgae researchers know empirically that they show adaptation to a strong light, there have been no quantitative reports on the temporal changes in the photophobic responses of *E*. *gracilis*; that is, how they become more tolerant to blue light over time. The effects of the circadian rhythm, the metabolic status of the cells, and diversity of cellular characteristics on the photophobic responses of *E*. *gracilis* are also unclear, because there is a lack of experimental data on changes in cell motion over time. Therefore, there is a need to understand fundamental aspects of changes in the photophobic responses of *E*. *gracilis* over time, and to identify the factors involved in these changes.

In this paper, we describe a time-course motion analysis of the photophobic responses of *E*. *gracilis* over several hours. Our main purpose of this research is not to identify specific molecules responsible for the temporal changes, but to quantify changes in swimming motion and discuss dominant factors involved in the adaptive temporal changes. We examined changes in motion induced by strong blue light under two different experimental conditions; spatially uniform periodic illumination, and spatially patterned constant illumination. The former revealed the photophobic motion of all cells, whereas the latter revealed how individual cells are capable of escaping from blue light. We observed four distinctive photophobic motions, and clarified experimentally that the ratio of these four responses depends on the metabolic status of the cells, rather than circadian rhythms, the cell cycle, or blue-light adaptation. We also show that, different from the photophobic responses and phototaxis of *C*. *reinhardtii*, the redox status of *E*. *gracilis* cells does not affect their photophobic responses largely. The observations show that the photophobic motion type of *E*. *gracilis* evolves from rotation to straightforward swimming, dominated mainly by the nutrient metabolic status of the cells. This leads to a hypothesis that the flagellar motion of *E*. *gracilis* in photophobic response is switching straightforward swimming and rotation according to the nutrient-rich metabolic status of the cells.

## Results and discussion

### Photophobic responses observed in micro-chamber

**Fig 1:**
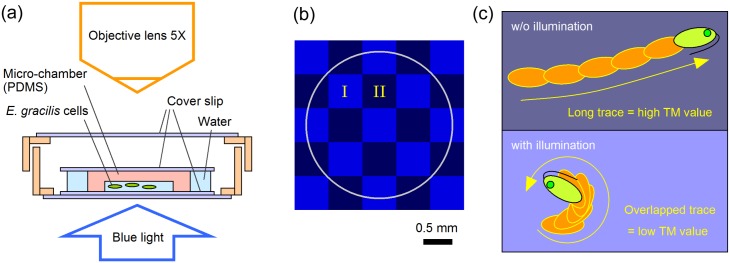
Experimental illustrations. (a) Cross-sectional illustration of experimental setup of micro-chamber containing *Euglena gracilis* cells. Micro-chamber with a depth of 140 μm was covered with a thin glass, and was enclosed in a dish. Blue light was illuminated from the bottom side of the dish. Cell motion was observed from the top through a microscopic lens (5×). (b) Blue-light checkerboard pattern of 580-μm squares used in experiment B. Circle indicates outer edge of micro-chamber. Brighter (darker) blue area was illuminated (non-illuminated), demoted as area I (II). All area I and II were periodically illuminated in experiment A. (c) Typical movement of *E*. *gracilis* with and w/o strong blue light illumination. The cells swim straightforward w/o illumination, yielding line-shape traces with smaller overlaps. They rotate under strong blue light, yielding spotty traces with larger overlaps. By counting the number of pixels of the traces in a specific area, their movements in the area are evaluated as one *TM* value. The *TM* value also depends on the number of the cells in the area of interest, i.e., the smaller (larger) number of the cells in a specific area results in a smaller (larger) *TM* value for the area.

The experiment setup of our system is given in Fig 1(a). The key aspect of the experimental configuration is that the photo-stimulus came from the bottom side of the dish, whereas the cells were confined in the micro-chamber and swimming horizontally. This means that the photo-induced movements we observed in our system were not phototaxis, i.e., photo-directional motions, but photophobic responses, i.e., non-directional motions, with which they try to escape from the strong light by changing their swimming direction randomly.

In this report, we conducted two types of experiments alternately (ABAB…); cyclic whole area illumination as experiment A, and constant checkerboard-pattern illumination for 17 min as experiment B. The checkerboard pattern in the experiment B is shown in Fig 1(b). We index the experiments A such as 4A_3, which represents the 3rd pulse in the experiment A at the 4th cycle. The illuminated (non-illuminated) area in the experiment B is denoted by area I (II), and thus 3B_I represents the cell distribution in the illuminated area in the experiment B at the 3rd cycle. The duration of the experiment A and B was 26 min each, and one cycle of the experiment AB was 53 min. The movements of the cells in a specific area were evaluated as trace momentum (*TM*–see Materials and methods for details of the *TM* calculation).

### Initial photophobic responses

**Fig 2:**
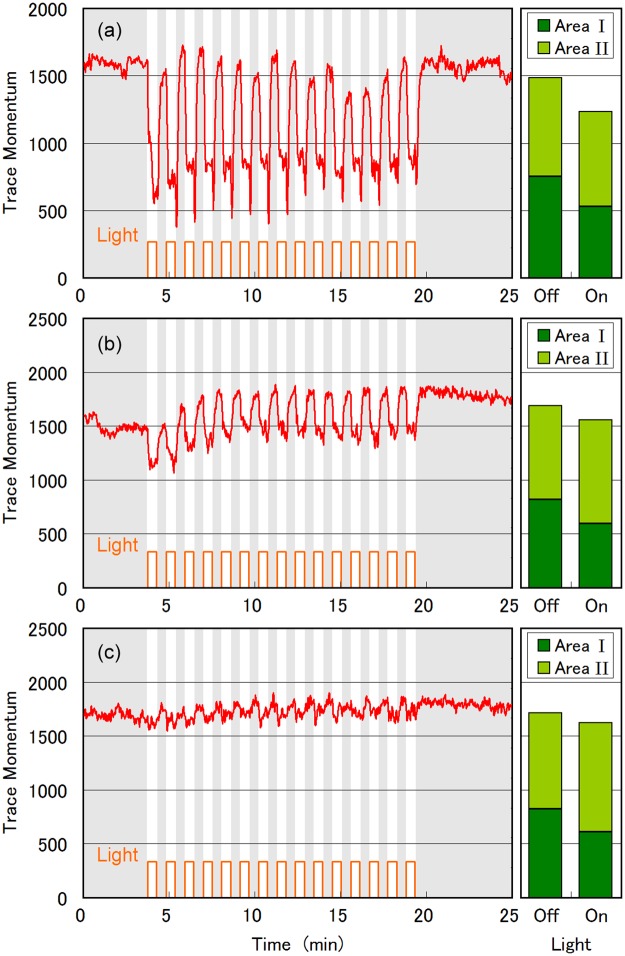
Photophobic responses as TM changes. Photophobic responses of *Euglena gracilis* observed by alternated repetition of experiments A and B. Experiment A: blue-light pulse illumination of whole area with 32-s on/32-off. Change in *TM* over time is plotted in left panels for first experiment (a), second experiment (b), and fourth experiment (c). Experiment B: blue-light checkerboard illumination pattern as shown in Fig 1(b) for 17 min out of total experiment time of 26 min. Bar graphs in right panels show *TM* for illuminated (non-illuminated) area I (II) averaged for blue-light on-duration (17 min) and off-duration (9 min).

**Fig 3:**
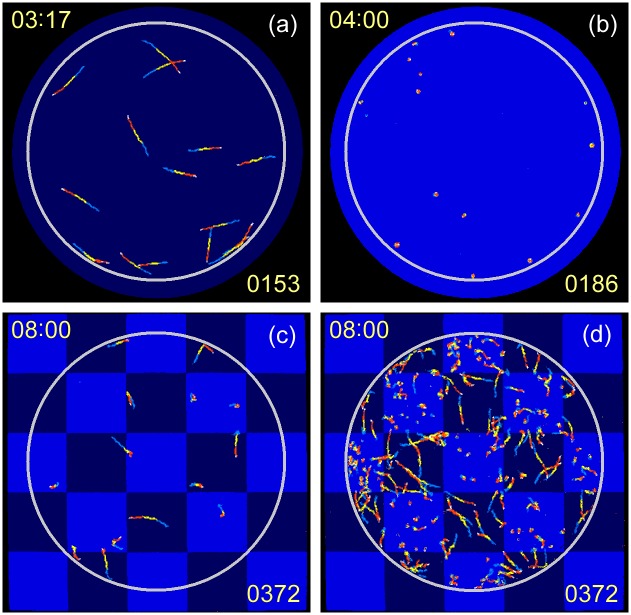
Swimming trace images observed for 4th cycle. Swimming traces observed for first experiment A before blue-light pulse at 197 s (a) and during first pulse at 240 s (b); and for first experiment B during blue-light checkerboard pattern illumination at 480 s for a smaller group of cells (c) and for a separate experiment with a larger group of cells (d). Traces were superimposed for a period of 3.9 s to show swimming movements, and coloured in the order blue, yellow, red, and white, to indicate swimming direction.

Fig 2(a) shows a typical example of the photophobic response observed in the experiment A. The whole area was illuminated with strong blue light, and we focused on a small cell population of 16 cells. There was a large decrease in *TM* when the cells were illuminated with blue light, confirming that blue light affected their swimming motion. The cells moved in straight lines without blue-light illumination (Fig 3(a)), but the swimming traces changed to small spots under blue-light illumination (Fig 3(b)). The video streaming of the trace images (S1 Movie) revealed that the cells rotated on-site. This change from straightforward swimming to on-site rotation occurred within 2.0 s, and the cells continuously rotated on-site during blue-light illumination for 32 s. On-site rotation was reported decades ago as the common step-up photophobic response of *E*. *gracilis* [19–22,25,26]. The cells resumed a straightforward swimming motion when the blue-light illumination ended, and the *TM* recovered (Fig 2(a)). Therefore, the decrease/recovery in *TM* corresponding to on/off illumination in Fig 2(a) was caused by the well-known photophobic response of *E*. *gracilis*, except for the downward spikes at the end of the blue-light illumination. These downward spikes in *TM* reflected the instant freezing behaviour of the cells upon termination of the blue light, which we have reported previously [27]. This switching behaviour between straightforward swimming and on-site rotation occurred repeatedly upon on/off blue-light illumination during the first experiment A (S1 Movie).

The *TM* values for the area I and II for the experiment 1B are plotted in the right panel in Fig 2(a). For the blue-light-off period in the experiment 1B, the *TM* separation ratio (defined as *TM*_*II*_/(*TM*_*I*_ + *TM*_*II*_)) was 0.50, indicating that the cells were equally distributed in areas I and II. When blue light was irradiated onto area I, the ratio increased to 0.57, because *TM*_*I*_ decreased due to the photophobic responses of the cells in area I. The trace image observed at 8.0 min (blue-light-on period) in the experiment 1B is shown in Fig 3(c). The cells swam straight forward in area II, but rotated on-site in area I. The distribution and motion of cells can be seen more clearly when a larger number of cells (approximately 200 cells) was used, as shown in Fig 3(d). The cell population in area I (II) was 57% (43%) (Fig 3(d)), indicating that the cells failed to escape from the illuminated area I. Some swimming cells in the non-illuminated area II accidentally moved into illuminated area I, and switched to on-site rotation as a result of the strong blue light. This resulted in a relatively larger cell population in area I. The *TM* separation ratio of *TM*_*II*_/(*TM*_*I*_ + *TM*_*II*_) was 0.49 (Fig 3(d)). The *TM* separation ratio was close to 0.5 because the *TM* for a single cell was larger for swimming cells with straightforward traces than for on-site rotating cells with spotty traces. The results of the experiment 1B showed that the photophobic on-site rotation response was ineffective for escaping from the illuminated area I.

### Evolution of photophobic responses

**Fig 4:**
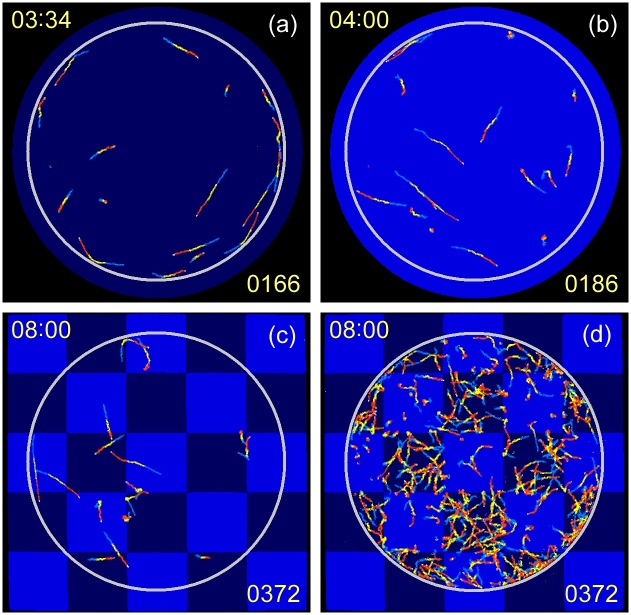
Swimming trace images observed for 4th cycle. Swimming traces observed for fourth experiment A before blue-light pulse at 214 s (a) and during first pulse at 240 s (b); and for fourth experiment B during blue-light checkerboard pattern illumination at 480 s for a smaller group of cells (c) and for a separate experiment with a larger group of cells (d). Traces were superimposed for a period of 3.9 s to show swimming movements, and coloured in the order blue, yellow, red, and white, to indicate swimming direction.

When experiments A and B were repeated alternately, different responses in *TM* were observed as shown in Fig 2(b) for the second experiment and in Fig 2(c) for the fourth experiment. The blue-light-induced decrease in *TM* was smaller in the experiment 2A (Fig 2(b)), and largely disappeared in the experiment 4A (Fig 2(c)). The swimming traces observed in the experiment 4A showed that 60%–80% of the cells did not rotate on-site, but showed distinctive swimming under blue-light illumination (Fig 4(b), and S2 Movie. Accordingly, the distribution and motion of the cells in the experiment 4B differed from those in the experiment 1B (Fig 4(c) and 4(d)). The cells escaped from the illuminated area I more effectively in the experiment 4B than in the experiment 1B, resulting in a larger cell population in area II than in area I. In the experiment 4B, the population of cells in area I (II) was 27% (73%) (Fig 4(d)). Correspondingly, the *TM* separation ratio of *TM*_*II*_/(*TM*_*I*_ + *TM*_*II*_) was 0.72.

**Fig 5:**
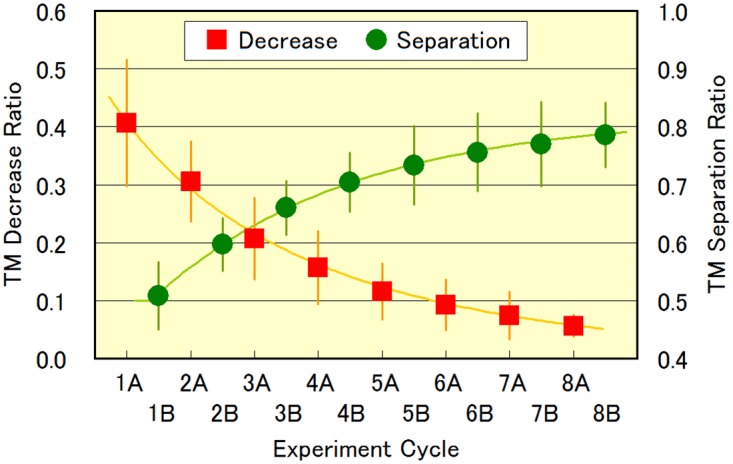
Temporal evolution of photophobic responses. Changes in photophobic responses in experiments A and in spatial separation in experiments B. Photophobic response in A was evaluated from *TM* during fourth blue-light pulse (*TM*_*on*_) and original *TM* before and after fourth blue-light pulse (*TM*_*off*_) as *TM* decrease ratio: (*TM*_*off*_—*TM*_*on*_)/*TM*_*off*_. Spatial separation was evaluated from *TM* for illuminated area (*TM*_*I*_) and non-illuminated area (*TM*_*II*_) as *TM* separation ratio: *TM*_*II*_/(*TM*_*I*_ + *TM*_*II*_). *TM* decrease ratio represents proportion of cells that changed swimming motion from straightforward swimming to on-site rotation. *TM* separation ratio represents how effectively cells escaped from blue-light illuminated area I and remained in non-illuminated area II. One experiment cycle corresponds to approximately one hour (55.5 min). We conducted the series of the experiment 11 times, and plotted the average values with their standard deviation as error bars.

Fig 5 shows the changes in the photophobic response over time (represented by a decrease in *TM*) in experiments A, and changes in the spatial separation over time (*TM* separation) in experiments B. The photophobic response in experiments A was evaluated from *TM* during the fourth blue-light pulse (*TM*_*on*_) and the original *TM* before and after the fourth blue-light pulse (*TM*_*off*_), as the *TM* decrease ratio (*TM*_*off*_—*TM*_*on*_)/*TM*_*off*_. The statistics for Fig 5 are given in S1 Table. The photophobic response represented by the *TM* decrease ratio was 0.41 for the experiment 1A, and decreased exponentially to 0.06 for the experiment 8A. At the same time, the spatial separation represented by the *TM* separation ratio increased from 0.51 to 0.79 throughout the experimental cycle. These changes over time revealed that the photophobic responses of the cells changed during the experimental cycle, with fewer cells showing on-site rotation as the cycles progressed. This improved the efficiency of escape from the blue-light illuminated area. From the dependence, we deduced the time scale of adaptive change of photophobic responses of *E*. *gracilis* as 2.9–3.1 h, i.e., the initial photophobic response was relaxed to 1/3 or less after approximately 3.0 h.

### Photophobic swimming motions

**Fig 6:**
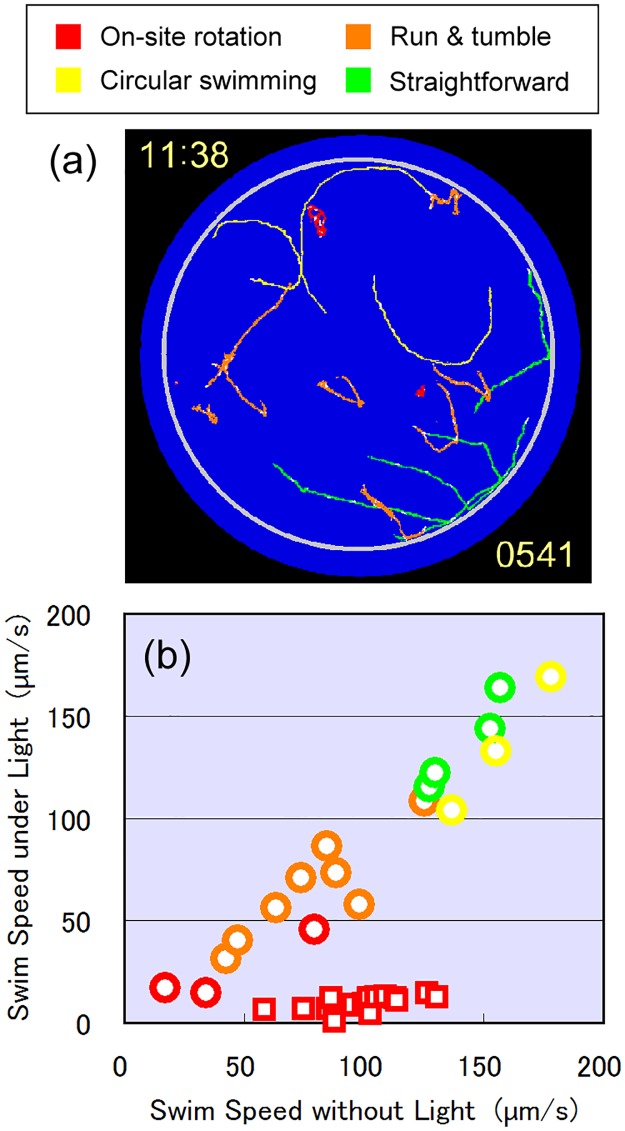
Photophobic motion types and swimming velocity. (a) Four types of photophobic swimming motion observed during eighth blue pulse in fourth experiment A. Subsequent swimming traces for 688–698 s were superimposed. Four distinct motions were observed: on-site rotation (red), running and tumbling (orange), circular swimming (yellow), and straightforward swimming (green). Each motion was categorized after observing motion continuity during whole period of eighth blue pulse (678–709 s). (b) Relationship between swimming speed without or with blue-light illumination and type of photophobic response motion during first blue-light pulse in first experiment A (squares) and during eighth blue-light pulse in fourth experiment A (circles). Type of photophobic response motion is indicated by colour: red (on-site rotation), orange (run and tumble), yellow (circular swimming), and green (straightforward swimming).

The changes in the photophobic swimming motions of cells were analyzed by tracking the swimming traces observed in experiments A. Fig 6(a) shows a typical example of swimming traces observed during the 4A_8 pulse. Eight subsequent traces are shown superimposed in Fig 6(a) to track the swimming motion of each cell. Four distinctive motions were observed; on-site rotation with limited spatial locomotion, running and tumbling (run/tumble) with occasional changes in direction, continuous swimming in a circle with a large diameter, and straightforward swimming (Fig 6(a)). On-site rotation is the conventional photophobic step-up response that most cells showed in the experiment 1A (Fig 3(b)). The run/tumble motion is a behaviour known as Levy flight [28,29], i.e., straightforward motion with occasional short tumbling to change the swimming direction [30]. The cells showing a run/tumble motion changed their swimming direction three to five times during the 32-s blue-light pulse. As far as we know, the response to swim in a large circle has not been reported as a photophobic response of *E*. *gracilis* previously. In this response, the cells swam in a circle with a large diameter (300–1200 μm), but resumed straightforward swimming when the blue light was terminated. The cells showing straightforward swimming (Fig 6(a)) showed no photophobic response to the blue light, at least during the 32-s blue-light pulse.

In some cases, we observed ambiguous intermediate motions between on-site rotation and run/tumble, between run/tumble and straightforward swimming, and between circular swimming and straightforward swimming. We categorized the motions with tumbling more than once during the 32-s blue-light pulse as run/tumble. The motions were categorized as circular swimming when the trace of the cell was more circular than straight. A small proportion of the cells (<10%) stopped moving during the experiments, or rotated on-site only during the blue-light pulse. These irregular cells were excluded from the motion analysis.

The swimming speed of the cells was strongly correlated with their photophobic motions. As shown in Fig 6(b), the photophobic response of the cells with a faster swimming speed (>120 μm/s) was mostly circular swimming or straightforward swimming, whereas that of cells with a moderate swimming speed (50–100 μm/s) was run/tumble. This correlation suggested that the metabolic status of the cells, which governs their locomotive activity, also determines the type of photophobic response motion. The swimming speed of the same cells was much more uniform in the experiment 1A (Fig 6(b)), ranging from 50 to 140 μm/s. This indicated that the cells changed their swimming speed and their photophobic response motion between the experiment 1A and 4A (a time difference of <3 h). Some cells lowered their swimming speed and rotated on-site as their photophobic response, whereas others increased their swimming speed over time, and showed three different types of photophobic response motions.

**Fig 7:**
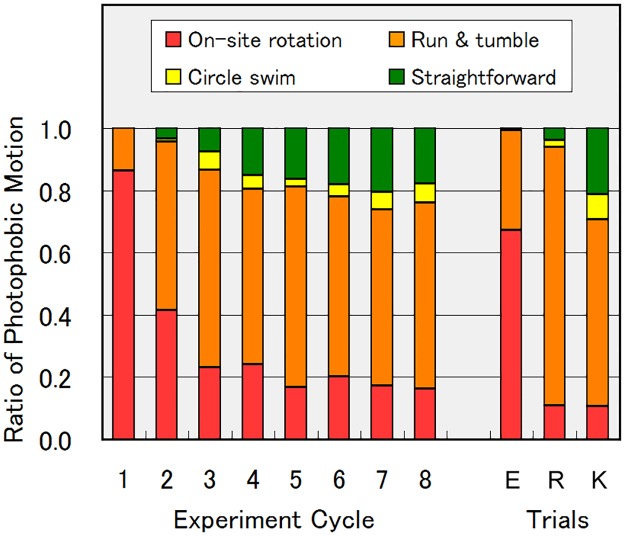
Evolution of photophobic motion types. Histogram of proportions of photophobic motion types. Left eight bars show changes in photophobic motion types over time from first to eighth experiments A (as at third blue-light pulse in each experiment). One experiment cycle corresponds to approximately one hour (55.5 min). Right three bars show first experiment A starting in the evening (marked E), after 6-h red light illumination (marked R), and after culture in 8KH:2CM medium (marked K), analyzed at third blue-light pulse. All histograms show values averaged from seven or eight experiments. The obtained data showed a standard deviation of 0.00–0.20 (0.08 in average).

The changes in the photophobic response motions over time are shown in Fig 7. In Fig 7, the proportions of cells showing each of the four photophobic response motions during the experiment nA_3 pulse (n = 1 to 8) are plotted. The statistics for Fig 7 are given in S2 Table. The proportion of cells rotating on-site was initially 90%, and decreased to 40% in 2A_3 pulse, and to 20% in 3A_3 pulse. The proportion of cells showing the run/tumble motion increased from 10% in 1A_3 to 50%–60% in 2A_3. The increase in the proportion of cells showing the circular swimming response was less than 10%. The proportion of cells showing the straightforward swimming response gradually increased throughout the experiment cycle and reached 20% in 5A_3. The average *TM* value for a single cell under blue light was 35, 69, 98, and 103, for on-site rotation, run/tumble, circular swimming, and straightforward swimming, respectively (Fig 6(a)). A higher proportion of cells showing on-site rotation results in a smaller *TM* in total. Therefore, the dependence of the *TM* decrease ratio on the experiment cycle in Fig 5 is attributed to changes in the types of photophobic response motion shown in Fig 7. At the same time, the run/tumble motion increased the chances of escape from the illuminated area in the experimental series B, leading to the increase in the *TM* separation ratio over time shown in Fig 5.

### Comparative experiments

The results showed that the photophobic responses of the cells changed over time, and the cells became more tolerant to blue-light illumination. The cells with the run/tumble response were better able to escape from the blue-light-illuminated area, whereas those with circular or straightforward swimming retained their ability to swim quickly even under blue light. In other words, the development of the photophobic response led to higher locomotive activity under blue light, which would be advantageous for survival in harsh conditions with strong blue light. Two questions arise from these results: how does the photophobic response develop, and what causes the variation in photophobic motion?

The factors that are most likely to be responsible for the development of photophobic responses are cell cycle, circadian rhythm, blue-light adaptation, and cellular metabolic status. The cells used in this study were cultured in microtubes under weak light (approximately 0.2 mW/cm^2^) during the daytime and in darkness at night. Cell multiplication was suppressed by the shortage of light energy under these conditions. When the cells were transferred into a closed micro-chamber (see Materials and methods for details of the micro-chamber) and illuminated with white light of 0.3–0.4 mW/cm^2^ for 8 h, the *TM* value and number of swimming cells decreased by 50%, and then increased to 150% as a result of cell multiplication. From this observation, we estimated that approximately 50% of the cells were synchronized in their cell cycle and circadian rhythm.

The effect of the natural cell cycle and circadian rhythm on the cells in the microtubes was evaluated by starting the experiments in the evening (approximately 5:30 pm), i.e., 8 hours behind the standard experiments. As shown in Fig 7, the ratio of photophobic responses in the experiment 1A in the evening (E in Fig 7) was similar to that in the morning experiment (experiment cycle 1 in Fig 7), although the proportion of cells rotating on-site was lower (67%). The large deviation of the ratio from those in the standard experiment cycles 3–8 (20%) indicated that the development of the photophobic response (a decrease in the proportion of cells showing on-site rotation from 90% to 20%) was not caused by the natural cell cycle or by the circadian rhythm of the cells.

The effect of adaptation to blue light was examined by repeating the same experimental series (A and B), but eliminating blue-light illumination from cycles 1 to 7. The ratio of photophobic responses in the experiment 8A of the above condition, i.e., after only red light illumination for approximately 6.5 h, is shown in Fig 7 (marked as R). The proportion of cells showing on-site rotation was 11%, lower than those in the standard experiment cycles 3 to 8 (20%). This result indicated that adaptation to blue light was not the dominant factor responsible for the reduction in the number of cells showing on-site rotation. Instead, this result revealed that red light illumination promoted the development of photophobic responses. Because the cells were illuminated with a strong red light of 12 mW/cm^2^ during the experimental cycles 1 to 7 (approx. 6.2 h) and blue light as well, their metabolic status increased as a result of photosynthesis. We consider that the improved metabolic status contributed to the increase in the run/tumble response instead of the on-site rotation response. The proportions of cells showing circular (2%) and straightforward swimming (4%) responses remained low in the absence of blue-light illumination (marked as R in Fig 7). This suggested that the repetition of blue-light illumination promoted the transition from the run/tumble response to the circular or straightforward swimming responses.

To clarify the effects of metabolic status on the development of the photophobic response, we added external nutrients to the culture medium to increase the nutrient-rich metabolic status of the cells. The cells with external nutrients were prepared by culturing cells in 80% nutrient-rich Koren-Hutner (KH) medium [31] + 20% CM medium [32], in which the cells can grow exponentially without light for photosynthesis. The cells multiplied in the mixed KH+CM medium for 1 week in the weak light. Their photophobic responses in the first experiment A are shown in Fig 7 (marked as K). Among these cells, 10% showed on-site rotation, 60% showed run/tumble, 8% showed circular swimming, and 21% showed straightforward swimming under blue light. These proportions were similar to those in the experiment 8A (Fig 7), supporting the hypothesis that more nutrient-rich metabolism in the cells leads to decreased on-site rotation, as in the case of 6-h red-light illuminated cells (marked R in Fig 7). The gene expression level of blue-light photoreceptor PAC was 10%–30% higher in KH-cultured cells than in CM-cultured ones, as estimated from fragments per kilobase of exon per million mapped fragments (FPKM) values. Although the cells cultured in KH medium may have more PAC proteins than those in CM medium, they were more tolerant to blue-light illumination. This suggests that the cellular metabolic status determines the photophobic motions more dominantly than the amount of PAC proteins. The addition of nutritious KH medium increases nutrients in the cells and improves the photo-tolerance (robustness) of the cells. We consider that the nutrient-rich status leads to a high energetic status with high ATP/ADP or NADH/NAD+ ratios, contribute to higher beating frequency of the flagellum and the photo-tolerance motions observed.

### Effect of oxidative stress

**Fig 8:**
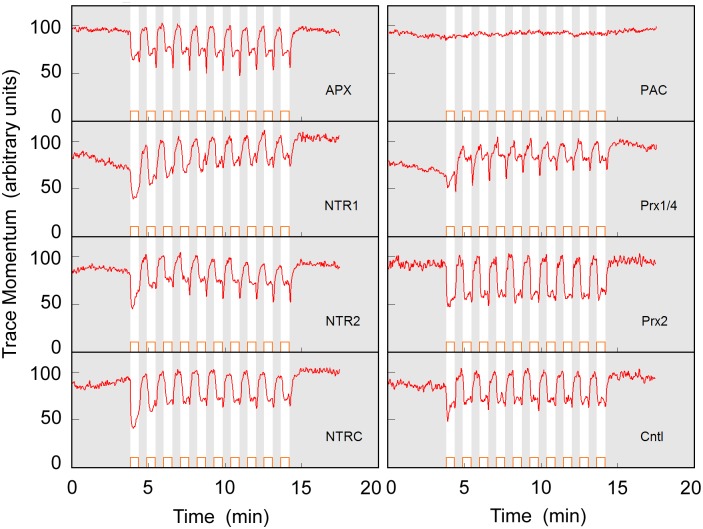
Photphobic responses of gene knock-down lines. Temporal change of *TM* induced by blue-light pulse illumination in gene knock-down lines. Except for *PAC*-knockdown line, variations in peak shape, depth, and spikes were attributed to uncontrollable fluctuation in cell preparation processes. Essential feature that *TM* decreases under a blue-light pulse is conserved for *APX*-, *NTR*-, and *Prx*-knockdown lines, showing that cell redox status does not strongly affect the photophobic responses of *Euglena gracilis*.

Wakabayashi et al. reported that the redox status of *C*. *reinhardtii* cells affected their photophobic responses [33] as well as phototaxis [34]. They observed that the cells showed flagellar beat frequency decrease, photophobic response duration increase, or positive phototaxis after treatment with reactive oxygen species (ROS), and vice versa after treatment with ROS quenchers [33,34]. On the possible analogy of the *C*. *reinhardtii* case, we consider that the redox status of *E*. *gracilis* cells may affect their photophobic responses, and investigated the redox status effect by using the RNA interference (RNAi) technique to knock-down genes encoding various redox-related enzymes. The genes selected for knock-down were those encoding APX [35] (ascorbate peroxidase), NTRs (NADPH-dependent thioredoxin reductases) [36], and Prxs (peroxiredoxins) [37], as well as that encoding the blue-light sensor protein PAC (photoactivated adenylyl cyclase) [38] for comparison. Representative responses of the four types of knock-down cells in the experiment A are shown in Fig 8. The statistics for Fig 8 are given in S3 Table. All of the knocked-down lines except the *PAC*-knockdown line showed similar responses to that of wild-type *E*. *gracilis* (Fig 2(a)). Although there were some variations in peak shape, depth, and spikes, the essential feature that *TM* decreased under a blue-light pulse was preserved in each knock-down line. Some variations in waveform in Fig 8, especially for NTR2 and Prx1/4, were caused by the slow recovery of on-site rotating cells, transient freezing at the timing of light-off [27], and the photo-induced activation of resting cells. The essential photophobic motions of these cells, such as on-site rotation or run/tumble, was well preserved for all RNAi cells except the case of PAC.

**Fig 9:**
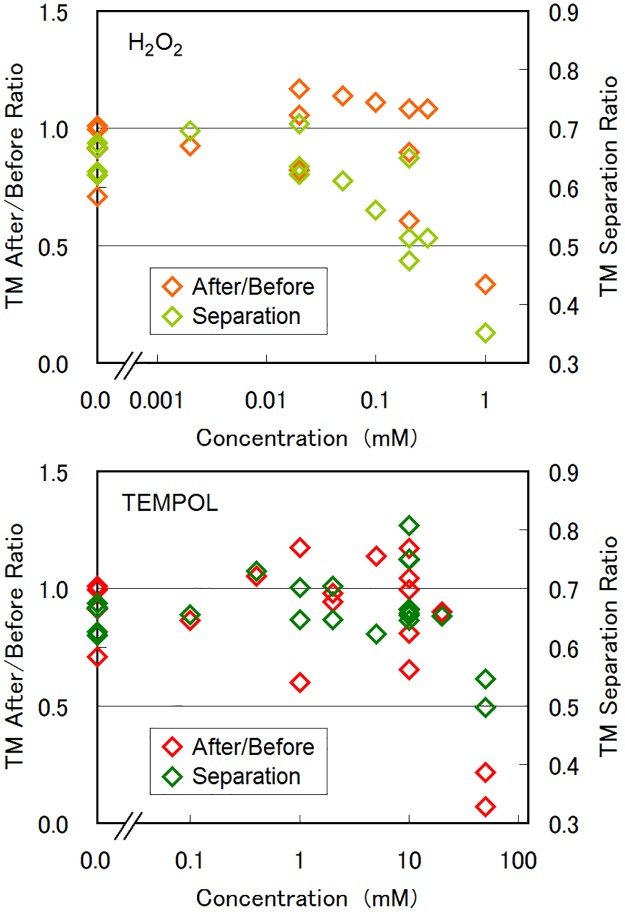
Effects of oxidative stress. Effect of H_2_O_2_ and TEMPOL on the swimming activity (*TM* after/before ratio) and photophobic response (*TM* separation ratio), examined by the experiment B. The swimming activity was evaluated by the ratio of average *TM* value after the checkerboard illumination over that before the illumination. The photophobic response was defined as *TM* separation ratio as that in Fig 5. At a higher H_2_O_2_ (TEMPOL) concentration of 1.0 mM (50 mM), the swimming activity of the cells was decreased dramatically. The photophobic response was not dominantly affected by H_2_O_2_ (TEMPOL) up to 300 μM (20 mM), although a slight decrease in *TM* separation ratio was observed for H_2_O_2_.

We also examined the effects of the environmental redox conditions on the photophobic responses of *E*. *gracilis* by adding the oxidative stress reagent H_2_O_2_ or the ROS-quenching reagent TEMPOL (4-hydroxy-2, 2, 6, 6-tetramethyl piperidine 1-oxyl) to the culture medium. Fig 9 shows the effect of the exogenous reagents of H_2_O_2_ and TEMPOL on the swimming activity and photophobic response, examined with the checkerboard illumination (experiment B). The statistics for Fig 9 are given in S4 Table. At a higher H_2_O_2_ (TEMPOL) concentration of 1.0 mM (50 mM), the swimming activity of the cells, defined as *TM* preservation ratio before/after checkerboard illumination, was decreased dramatically, showing that a strong stress of redox unbalance reduces the swimming capability of the cells. Neither of these reagents significantly affected the photophobic behaviour of the cells up to 300-μM H_2_O_2_ or up to 20-mM TEMPOL. A slight decrease in *TM* separation ratio observed for H_2_O_2_ dose of 100–300 μM may suggest that the cells fell into on-site rotation at illuminated areas more easily.

These results showed that the cellular redox status was not a dominant factor affecting the photophobic responses of *E*. *gracilis*. No photophobic response was observed for the *PAC-*knock-down cells, indicating that PAC is the only blue-light sensor involved in the photophobic responses of *E*. *gracilis*. Since PAC molecules localize close to the base of the flagellum in *E*. *gracilis* cells, the cAMP produced by blue-light-activated PAC molecules might regulate flagellum movement regardless of the cellular redox status.

### Divergence in photophobic response

The temporal type changes in photophobic swimming motions from on-site rotation to run/tumble and final straightforward swimming can be explained by assuming the variation of switching frequency between rotational and straightforward flagellar motions. The cells show on-site rotation when the flagellar motion is rotational and not switched back to straightforward motion within a time scale of a couple of ten second or more. They show run/tumble (straightforward swimming) when the straightforward flagellar motion is switched intermittently to rotational motion with a time scale of a couple of seconds (a couple of ten seconds or more). The circular swimming may be caused by incomplete switching of flagellar motions. We speculate that the adaptive temporal changes observed in the time course of hours are evoked by a threshold increase for triggering the rotational flagellar motion, or by enhancement in flagellar switching back to straightforward motion, with a metabolic status change as photosynthesis proceeds. This hypothesis explains reasonably the observed temporal evolution of photophobic motion type. However, it is hard to confirm the hypothesis experimentally at the moment, since the exact molecule that accumulates in the cell after several rounds of light exposure, inhibits on-site rotation, and facilitates run/tumble behaviour, cannot be identified easily from a huge number of protein molecules in the cells even with a modern protein analysis technique.

We consider that the redox status does not affect dominantly the photophobic responses of *E*. *gracilis*, at least in the experiments made in this study, since the knock-down of enzyme codes should bring redox unbalance in the cells, as evidenced by H_2_O_2_ accumulation in the APX-RNAi strains [35]. The consideration is also supported by the fact that the addition of exogenous reagents of H_2_O_2_ (up to 300 μM) or TEMPOL (up to 20 mM) evoked no specific change in the photophobic response of *E*. *gracilis*. When the dose of H_2_O_2_ was increased to 1.0 mM, the cell fell into continuous rotation [12] with/without blue light. When the dose of TEMPOL was increased to 50 mM, the cell movements were decreased gradually. This indicates that a strong stress of redox unbalance reduces the swimming capability of the cells rather than induces the type change of the photophobic motions.

The photophobic responses observed at the 1st experiment A were rather uniform on-site rotation as shown in Fig 3(b), whereas the same cells exhibited four different types of photophobic responses. We consider that the deviation in the response type is due to the difference of cell characteristics, such as the photosynthesis efficiency, photosensitivity, strength in flagellum beating, or nutrition accumulation/consumption ratio. For an example, the photosynthesis efficiency depends on the number of chloroplasts, which differs cell by cell. As well, photosensitivity for blue light depends on the number of PAC proteins and their reaction efficiency. Due to such cell–cell variations, the metabolic status in each cell developed with variation, resulting in divergence in the photophobic response. It is noteworthy that the ratio of four photophobic responses in Fig 7 shows saturation after the 3rd experiment cycle, and not converged to one single response. This indicates that the cell personality is widely affected to the photophobic response even after enough photosynthesis of six hours. We consider that the divergence in photophobic response is one of the important survival strategies of *E*. *gracilis*.

## Conclusion

In conclusion, we examined temporal changes in the photophobic responses of *E*. *gracilis* under alternating blue-light pulse illumination and spatially patterned blue-light illumination. We observed four photophobic motions: on-site rotation with very limited spatial locomotion; running and tumbling (run/tumble) with occasional changes in the swimming direction; continuous swimming in a circle with a large diameter (up to 1.2 mm); and unaffected straightforward swimming. At the beginning of blue-light illumination, the photophobic response was mainly on-site rotation (87%), whereas the proportion of other motions, especially run/tumble, increased to 55%–65% within 1–2 h. A strong correlation was found between the photophobic response types and the swimming speed of the cells, i.e., the cells with higher swimming speeds tended to exhibit higher robustness to a strong light. We deduced that photosynthesis during these experiments improved the metabolic status of the cells, and the cells with a nutrient-rich metabolic status did not rotate on-site, which is ineffective for escaping from harsh blue light. In other words, the cells became more tolerant to blue-light illumination by improving their metabolic status and changing their photophobic motion. Although the cellular redox status is known to be an important factor in the photophobic responses and phototaxis of *C*. *reinhardtii*, it did not affect the photophobic responses of *E*. *gracilis*, as evidenced by the unchanged responses of the lines with knocked-down expression of redox-related genes. The fact deduced from our study is that the nutrient-rich cells have higher swimming speeds and higher robustness to a strong light. It leads to the hypothesis that the flagellar motion of *E*. *gracilis* is controlled comparatively (with the manner of switching frequency modulation) between straightforward swimming and rotation according to the nutrient-rich metabolic status of the cells.

## Materials and methods

### Cell culture and micro-chamber confinement

We used the microalga *Euglena gracilis* (Z-strain), which was maintained in Cramer–Myers’ (CM) medium as suspensions in 1-mL microtubes at room temperature. The microtubes were illuminated with a weak light of 0.2 mW/cm^2^ for approximately 10 h daily (8:30 am–7:30 pm), and in the dark at night. The cells in the microtubes were autotrophic with photosynthesis under the weak light, but their multiplication was suppressed by the low light level that was close to the light compensation point. This type of cell culture in microtubes is labour-free, cost-effective, readily transportable, and suitable for applications requiring only small volumes of cells.

A key aspect of this study was to observe the photophobic responses of a group of cells repeatedly. For this purpose, we transferred a small volume of cells from the microtubes and confined them in a closed, circular polydimethylsiloxane (PDMS) micro-chamber (diameter, 2.49 mm; depth, 140 μm). The cell suspension (0.7 μL, containing 20–200 cells) was covered with a glass slip inside the micro-chamber, allowing us to observe their photophobic responses repeatedly. The micro-chamber chip was contained in a glass-top and -bottom dish (Fig 1(a)).

### Photo-illumination and swimming trace analysis

Two types of experiments (A and B) were performed repeatedly on the same cells confined in the micro-chamber. Experiment A was spatially uniform periodic illumination, where the whole area of the micro-chamber was illuminated periodically by strong blue light with 15 repeats of light 32-s on and 32-s off. Experiment B was spatially patterned constant illumination, where blue light was illuminated in a checkerboard pattern of 580-μm squares (Fig 1(b)) for 17 min constantly. Experiments A and B were applied alternately eight times each for the same group of cells (i.e., ABABAB…). Experiments A and B were 28-min long. The first experiment A was started in the morning (9:30 am–10:00 am).

As reported in our previous paper [39,40], the illumination system was an optical feedback system consisting of a liquid-crystal (LC) projector (PT-VX400, Panasonic, Osaka, Japan) connected to a personal computer (MG/D70N, Fujitsu, Tokyo, Japan). The light emitted from the LC projector was reduced in size and illuminated onto the micro-chamber from the bottom, as illustrated in Fig 1(a). The intensity of blue light (430–520 nm) was typically 20 mW/cm^2^ (approximately 880 μmol/m^2^s). Red light (570–700 nm, 12 mW/cm^2^, approximately 460 μmol/m^2^s) was illuminated onto the micro-chamber constantly to observe cells’ swimming motions.

The swimming motion (trace) was extracted from video images acquired using a video camera (IUC-200CK2, Trinity, Gunma, Japan) mounted on an optical microscope (BX51, Olympus, Tokyo, Japan) with a 5× objective lens. By differentiating, thresholding, and superimposing the images, the extracted swimming traces were visualized with a refreshing rate of 0.77 fps. The swimming activity of the cells was evaluated by counting the number of pixels of the swimming traces in the illuminated/un-illuminated areas in trace images. We defined the spatial sum of swimming trace pixels as *trace momentum* (*TM*). When the cell swim straightforward (rotate on-site), the traces of a single cell have a smaller (larger) overlap, resulting in a larger (smaller) *TM* value, as shown in Fig 1(c). The *TM* value also depends on the number of the cells in the area of interest, i.e., the smaller (larger) number of the cells in a specific area results in a smaller (larger) *TM* value for the area. Further details of our experiments were provided in our previous reports [39,40].

### Oxidative stress experiments

We also examined the effects of redox-related enzyme knock-down on the photophobic response of *E*. *gracilis*. The genes encoding APX, NTRs (NTR1, NTR2, NTC), Prxs (Prx1/4 = Prx1 and 4, Prx2, Prx3), and the blue-light photoreceptor protein PAC for comparison, were individually knocked-down using the RNAi technique. For RNAi, double-stranded RNA (dsRNA) was synthesized using a MEGAscript RNAi Kit (Life Technologies, Carlsbad, CA) and PCR-produced cDNA templates. The dsRNA was introduced into the cells by electroporation (NEPA21, Nepa Gene, Chiba, Japan). Each gene was knocked down by suppressing its corresponding mRNA in the cells. The cells resuspended in CM medium (approximately 5×10^6^ cells in 100 μL) were electroporated with 15 μg of dsRNA and grown for 14 days in a fresh CM medium for restoration. Details of the gene knock-down process and cDNA sequences were provided in our previous reports [36,37], or Iseki's reports for PAC [38,41]. The suppression of individual gene expression in each knock-down cells was confirmed by RT-PCR analysis [36,37]. The correlation between mRNA-abundance and protein abundance has been reported previously [42].

## Supporting information

S1 Movie Video streaming of trace images of initial photophobic responses. Trace image movie of photophobic responses of *E*. *gracilis* cells observed at the experiment 1A, corresponding to Fig 2(a). When the whole area was illuminated by a strong blue light, all cells swimming in straight lines changed instantly their motion to on-site rotation. Their motion switched back when the light was terminated.
(MOV)

S2 Movie Video streaming of trace images of maturaed photophobic responses. Trace image movie of photophobic responses of *E*. *gracilis* cells observed at the experiment 4A, corresponding to Fig 2(c). Compared to BluePulse_1.mov, the photophobic responses were not only on-site rotation, but run and tumble, circular swimming, or straightforward swimming.
(MOV)

S1 Table Statistics for the experiments of Fig 5. Column A: experiment cycle, B: number of experiments, C: averaged number of cells, D: averaged *TM* per cell, E: averaged *TM* decrease ratio, and F: averaged *TM* separation ratio. Second number in column such as C1 and C2 represents average and standard deviation, respectively.
(XLS)

S2 Table Statistics for the experiments of Fig 7. Column A: experiment cycle/mark, B: number of experiments, C: averaged number of cells, D: averaged *TM* per cell, E: averaged proportion of on-site rotation, F: averaged proportion of run/tumble, G: averaged proportion of circular swimming, and H: averaged proportion of straightforward swimming. Second number in column such as C1 and C2 represents average and standard deviation, respectively.
(XLS)

S3 Table Statistics for the experiments of Fig 8. Column A: RNAi index, B: number of experiments, C: averaged number of cells, D: averaged *TM* per cell, and E: averaged *TM* decrease ratio. Second number in column such as C1 and C2 represents average and standard deviation, respectively.
(XLS)

S4 Table Statistics for the representative experiments of Fig 9. Column A: exogenous reagents and concentrations, B: number of experiments, C: averaged number of cells, D: averaged *TM* per cell, and E: swimming activity (averaged *TM* after/before ratio), F: averaged *TM* separation ratio. Second number in column such as C1 and C2 represents average and standard deviation, respectively.
(XLS)

## References

[1] Inui H, Miyatake K, Nakano Y, Kitaoka S. Wax ester fermentation in Euglena gracilis. FEBS Lett. 1982; 13:89–93. 10.1002/1873-3468.13276 PMC6587861 30328102

[2] Yamane Y, Utsunomiya T, Watanabe M, Sasaki K. Biomass production in mixotrophic culture of Euglena gracilis under acidic condition and its growth energetics. Biotechnol Lett. 2001; 23:1223–1228.

[3] Rodríguez-Zavala JS, Ortiz-Cruz MA, Mendoza-Hernández G, Moreno-Sánchez R. Increased synthesis of α-tocopherol, paramylon and tyrosine by Euglena gracilis under conditions of high biomass production. J Appl Microbiol. 2010; 109:2160–2172. 10.1111/j.1365-2672.2010.04848.x 20854454

[4] Siaut M, Cuiné S, Cagnon C, Fessler B, Nguyen M, Carrier P, et al Oil accumulation in the model green alga Chlamydomonas reinhardtii: characterization, variability between common laboratory strains and relationship with starch reserves. BMC Biotechnol. 2011; 11:7 10.1186/1472-6750-11-7 21255402 PMC3036615

[5] Berteotti S, Ballottari M, Bassia R. Increased biomass productivity in green algae by tuning non-photochemical quenching. Sci Rep. 2016; 6:21339 10.1038/srep21339 26888481 PMC4758054

[6] Kajikawa M, Kinohira S, Ando A, Shimoyama M, Kato M, Fukuzawa H. Accumulation of squalene in a microalga Chlamydomonas reinhardtii by genetic modification of squalene synthase and squalene epoxidase genes. PLoS ONE. 2015; 10:e0120446 10.1371/journal.pone.0120446 25764133 PMC4357444

[7] Schulz-Raffelt M, Chochois V, Auroy P, Cuiné S, Billon E, Dauvillée D, et al Hyper-accumulation of starch and oil in a Chlamydomonas mutant affected in a plant-specific DYRK kinase. Biotechnol Biofuels. 2016; 9:55 10.1186/s13068-016-0469-2 26958078 PMC4782384

[8] Ogawa T, Tamoi M, Kimura A, Mine A, Sakuyama H, Yoshida E, et al Enhancement of photosynthetic capacity in Euglena gracilis by expression of cyanobacterial fructose-1,6-/sedoheptulose-1,7-bisphosphatase leads to increases in biomass and wax ester production. Biotechnol Biofuels. 2015; 8:80 10.1186/s13068-015-0264-5 26056534 PMC4459067

[9] Yamada K, Kazama Y, Mitra S, Marukawa Y, Arashida R, Abe T, et al Production of a thermal stress resistant mutant Euglena gracilis strain using Fe-ion beam irradiation. Biosci Biotechnol Biochem. 2016; 14:1–7. 10.1080/09168451.2016.1171702 27075598

[10] Rawat I, Ranjith Kumar R, Mutanda T, Bux F. Biodiesel from microalgae: A critical evaluation from laboratory to large scale production. Appl Energy. 2013; 103:444–467.

[11] Bertolini D, Gualtieri P. Euglena Gracilis Case: A Real Biosensor In: Barsanti L, Evangelista V, Gualtieri P, Passarelli V, Vestri S, editors. NATO Science Series 96, Molecular Electronics: Bio-sensors and Bio-computers. Springer Netherlands; 2003 pp. 389–399.

[12] Ozasa K, Lee J, Song S, Hara M, Maeda M. Gas/liquid sensing via chemotaxis of Euglena cells confined in an isolated micro-aquarium. LabChip. 2013; 13:4033–4039. 10.1039/c3lc50696g 23934095

[13] Rechnitz GA, Ho MY. Biosensors based on cell and tissue material. J Biotechnol. 1990; 15:201–217. 1366674 10.1016/0168-1656(90)90027-9

[14] Ozasa K, Lee J, Song S, Hara M, Maeda M. Implementation of microbe-based neurocomputing with Euglena cells confined in microaquariums. Int J Unconventional Comput. 2011; 7:481–499.

[15] Bonnet J, Yin P, Ortiz ME, Subsoontorn P, Endy D. Amplifying genetic logic gates. Science. 2013; 340:599–603 10.1126/science.1232758 23539178

[16] Ozasa K, Lee J, Song S, Hara M, Maeda M. Autonomous Pattern Formation of Micro-organic Cell Density with Optical Interlink between Two Isolated Culture Dishes. Artificial Life. 2015; 21:234–246. 10.1162/ARTL_a_00159 25622016

[17] Ozasa K, Lee J, Song S, Hara M, Maeda M. Euglena-based neurocomputing with two-dimensional optical feedback on swimming cells in micro-aquariums. Appl Soft Comput. 2013; 13:527–538.

[18] Ozasa K, Lee J, Song S, Hara M, Maeda M. Analog Feedback in Euglena-Based Neural Network Computing-Enhancing Solution-Search Capability through Reaction Threshold Diversity among Cells. Neurocomput. 2014; 140:291–298.

[19] Wolken JJ, Shin E. Photomotion in Euglena gracilis I. Photokinesis II. Phototaxis. J Eukaryotic Microbiol. 1958; 5:39–46.

[20] Diehn B. Phototactic response of Euglena to single and repetitive pulses of actinic light: orientation time and mechanism. Exp Cell Res. 1969; 56:375–381. 5824457 10.1016/0014-4827(69)90027-5

[21] Häder D-P, Colombetti G, Lenci F, Quaglia M. Phototaxis in the flagellates, *Euglena* gracilis and Ochromonas danica. Arch Microbiol. 1981; 130:78–82.

[22] Doughty MJ. A kinetic analysis of the step-up photophobic response of the flagellated alga Euglena gracilis in culture medium. J Photochm Photobiol B: Biol. 1991; 9:75–85.

[23] Goto K, Laval-Martin DL, Edmunds LN Jr. Biochemical modeling of an autonomously oscillatory circadian clock in Euglena. Science. 1985; 228:1284–1288. 2988128 10.1126/science.2988128

[24] Hagiwara S, Bolige A, Zhang Y, Takahashi M, Yamagishi A, Goto K. Circadian Gating of Photoinduction of Commitment to Cell-cycle Transitions in Relation to Photoperiodic Control of Cell Reproduction in Euglena. Photochem Photobiol. 2002; 76:105–115. 12126300 10.1562/0031-8655(2002)076<0105:cgopoc>2.0.co;2

[25] Diehn B. Phototaxis and sensory transduction in *Euglena*. Science. 1973; 181:1009–1015. 4199225 10.1126/science.181.4104.1009

[26] Matsunaga S, Takahashi T, Watanabe M, Sugai M, Hori T. Control by Ammonium Ion of the Change from Step-Up to Step-Down Photophobically Responding Cells in the Flagellate Alga Euglena gracilis. Plant Cell Physiol. 1999; 40:213–221.

[27] Ozasa K, Lee J, Song S, Maeda M. Transient freezing behavior in photophobic responses of Euglena gracilis investigated in a microfluidic device. Plant Cell Physiol. 2014; 55:1704–1712. 10.1093/pcp/pcu101 25074906

[28] Metzler R, Chechkin AV, Gonchar VY, Klafter J. Some fundamental aspects of Lévy flights. Chaos, Solitons & Fractals. 2007; 34:129–142.

[29] Matthäus F, Jagodič M, Dobnikar J. E. coli Superdiffusion and Chemotaxis—Search Strategy, Precision, and Motility. Biophys J. 2009; 97:946–957. 10.1016/j.bpj.2009.04.065 19686641 PMC2726325

[30] Colombetti G, Lenci F, Diehn B. Responses to photic, chemical, and mechanical stimuli In: Buetow DE, editor. The Biology of Euglena: Physiology. Vol. 3 Academic Press; 1982 pp. 171–186.

[31] Koren LE, Hutner SH. High yielding media for photosynthesising Euglena gracilis. J Protozool. 1967; 14(Suppl):17.

[32] Cramer M, Myers J. Growth and photosynthetic characteristics of. *Euglena gracilis*. Arch Mikrobiol. 1952; 17:384–402.

[33] Wakabayashi K, King SM. Modulation of Chlamydomonas reinhardtii flagellar motility by redox poise. J Cell Biol. 2006; 173:743–754. 10.1083/jcb.200603019 16754958 PMC3207151

[34] Wakabayashi K, Misawa Y, Mochiji S, Kamiya R. Reduction-oxidation poise regulates the sign of phototaxis in Chlamydomonas reinhardtii. Proc Nat Acad Sci. 2011; 108:11280–11284. 10.1073/pnas.1100592108 21690384 PMC3131381

[35] Ishikawa T, Tajima N, Nishikawa H, Gao Y, Rapolu M, Shibata H, et al Euglena gracilis ascorbate peroxidase forms an intramolecular dimeric structure: its unique molecular characterization. Biochem J. 2010; 426:125–134. 10.1042/BJ20091406 20015051

[36] Tamaki S, Maruta T, Sawa Y, Shigeoka S, Ishikawa T. Biochemical and physiological analyses of NADPH-dependent thioredoxin reductase isozymes in Euglena gracilis. Plant Sci. 2015; 236:29–36. 10.1016/j.plantsci.2015.03.016 26025518

[37] Tamaki S, Maruta T, Sawa Y, Shigeoka S, Ishikawa T. Identification and functional analysis of peroxiredoxin isoforms in Euglena gracilis. Biosci Biotechnol Biochem. 2014; 78:593–601. 10.1080/09168451.2014.890037 25036955

[38] Iseki M, Matsunaga S, Murakami A, Ohno K, Shiga K, Yoshida K, et al A blue-light activated adenylyl cyclase mediates photoavoidance in *Euglena gracilis*. Nature. 2002; 415:1047–1051. 10.1038/4151047a 11875575

[39] Ozasa K, Lee J, Song S, Hara M, Maeda M. Two-dimensional optical feedback control of *Euglena* confined in closed-type microfluidic channels. LabChip. 2011; 11:1933–1940. 10.1039/c0lc00719f 21491041

[40] Ozasa K, Won J, Song S, Maeda M. Autonomous oscillation/separation of cell density artificially induced by optical interlink feedback as designed interaction between two isolated microalgae chips. Sci Rep. 2016; 6:24602 10.1038/srep24602 27098710 PMC4838927

[41] Ntefidou M, Iseki M, Watanabe M, Lebert M, Häder D-P. Photoactivated Adenylyl Cyclase Controls Phototaxis in the Flagellate Euglena gracilis. Plant Physiol. 2003; 133:1517–1521. 10.1104/pp.103.034223 14630964 PMC300708

[42] Yoshida Y, Tomiyama T, Maruta T, Tomita M, Ishikawa T, Arakawa K. De novo assembly and comparative transcriptome analysis of Euglena gracilis in response to anaerobic conditions. BMC Genomics. 2016; 17: 182 10.1186/s12864-016-2540-6 26939900 PMC4778363

